# Brain disposition of α-Synuclein: roles of brain barrier systems and implications for Parkinson’s disease

**DOI:** 10.1186/2045-8118-11-17

**Published:** 2014-07-31

**Authors:** Christopher A Bates, Wei Zheng

**Affiliations:** 1 School of Health Sciences, Purdue University, 550 Stadium Mall Drive, HAMP 1173, West Lafayette, IN 47907, USA

**Keywords:** Parkinson’s disease, α-Synuclein, Blood–brain barrier, Blood-cerebrospinal fluid barrier, Neurons

## Abstract

Parkinson’s disease (PD) is a neurodegenerative disorder characterized by the accumulation of α-Synuclein (a-Syn) into Lewy body inclusions and the loss of dopaminergic neurons in the substantia nigra (SN). Accumulation of a-Syn can induce a progressive, cyclical pathology that results in the transmission of toxic, aggregated a-Syn species to healthy neurons, leading to further neurodegeneration such as occurs in PD. The blood–brain barrier (BBB) and blood-cerebrospinal fluid (CSF) barriers (BCSFB) are responsible for regulating the access of nutrients and other molecules to the brain, but very little is known about their regulatory roles in maintaining the homeostasis of a-Syn in the CSF and brain parenchyma. This review analyzes the current literature reports on the transport of a-Syn by various brain cell types with a particular focus on the potential transport mechanisms of a-Syn at the BBB and BCSFB. The indication of altered a-Syn transport by brain barriers in PD pathoetiology and the perspectives in this research area are also discussed.

## Review

### Introduction

Parkinson’s disease (PD) is the second most common neurodegenerative disease after Alzheimer’s disease and typically affects those above the age of 60 [1]. PD is primarily defined by symptoms of motor impairment, i.e., resting tremor, rigidity, hypokinesia, and postural instability. Psychiatric problems (i.e. depression) and cognitive deficits are additional symptoms that have been associated with PD [2]. PD occurs in two general forms: sporadic and familial (genetic). The former occurs due to environmental exposure to a variety of toxicants that interact with vulnerable genes, and the latter involves genetic factors, e.g. point mutations and truncations that can occur in a variety of proteins [2]. Surviving neurons possess intra-cytoplasmic inclusions called Lewy bodies (LB) and/or Lewy neurites (LN) [3]. While these inclusions are present in the substantia nigra in the later stages of PD development (i.e. when PD can be clinically diagnosed), the inclusions have been shown to appear in other structures of the brainstem, e.g. anterior olfactory structures and the medulla oblongata [3] prior to their appearance at the substantia nigra. Furthermore, LBs/LNs are also present in the cerebral cortex in the final stages of PD [3]. These findings show that while the substantia nigra may be the prominent site of neurodegeneration, the dysfunction of α-synuclein (a-Syn) and the production of LBs/LNs begin elsewhere in the brain. This clearly indicates that the pathology contributing to the production of LBs/LNs propagates to other regions of the brain. An integral characteristic of LBs and LNs is that they are mainly comprised of the protein a-Syn [4]. The pathology of this protein is a hallmark of many neurological disorders including PD (e.g., multiple system atrophy, dementia with Lewy bodies, etc.) that have been grouped into a class of disorders called a-synucleinopathies.

The a-Syn protein is a small (~14.5 kDa), naturally unfolded protein expressed in a variety of cell types including neurons. The wild-type function of a-Syn is unknown, but a variety of functions have been proposed in recent literature. Potential functions of a-Syn at the neuronal synapse include vesicular stabilization [2], synaptic pool maintenance [5], regulation of dopamine synthesis [6], and a potential role in synaptic plasticity [7]. Additionally, an a-Syn-knockout murine model shows compromised complex I/III activity at the electron transport chain and changes in lipid composition of mitochondrial membranes [8]. The aggregation of a-Syn not only compromises synaptic functionality, but also the energy production at the mitochondria, which is clearly essential for all cell types [9]. These findings show that a-Syn is potentially involved in a variety of critical cellular processes in neurons. Unfortunately, the function of a-Syn in other cell types has yet to be elucidated.

Aggregation of a-Syn and ensuing development of a-Syn fibrils precede the development of Lewy bodies that contain highly aggregated a-Syn molecules. The most toxic species of a-Syn are the soluble, oligomeric species that are initially formed by the aggregation of a-Syn monomers [10]. The oligomeric species can then develop into larger, mature, insoluble fibrils of a-Syn that eventually assimilate into the insoluble, cytoplasmic LBs and LNs present in surviving neurons [10]. The process of a-Syn aggregation can be induced in a variety of pathways that range from mutations in various proteins including a-Syn to synaptic oxidative stress. More specifically, various mutations of a-Syn including point mutations (e.g. A53T, A30P, E46K), truncation, duplication, or triplication can result in the loss of wild-type function and an increased propensity to aggregate into fibrils [2,10,11]. Additionally, the overexpression of a-Syn in animal models has shown an increased a-Syn expression and the manifestation of PD-like symptoms with respect to controls [12-14].

Little is known about how a-Syn is regulated in the brain. Previous research has shown that wild-type and toxic species of a-Syn can be released by neurons to the interstitial fluid (ISF), which merges with the cerebrospinal fluid (CSF) [15-17]. While most molecules are cleared from the CSF by the choroid plexus or by drainage from the subarachnoid spaces to the blood [18,19], the clearance of aggregated a-Syn via the CSF remains unknown. It is possible that the blood-CSF barrier (BCSFB) in the choroid plexus may play a role in maintaining healthy a-Syn levels in the central milieu. In this review, the concepts and main functions of brain barrier systems, i.e., the blood–brain barrier (BBB) and BCSFB will be introduced. The current understanding on how a-Syn is transported by the neurons is then discussed, which is followed by a review of the current understanding of a-Syn transport by brain barriers and possible mechanisms underlying a-Syn transport. Finally, the indication of altered a-Syn transport by brain barriers in PD pathoetiology and the perspectives in this research area are discussed.

### The blood–brain barrier and blood-CSF barrier

**Figure 1:**
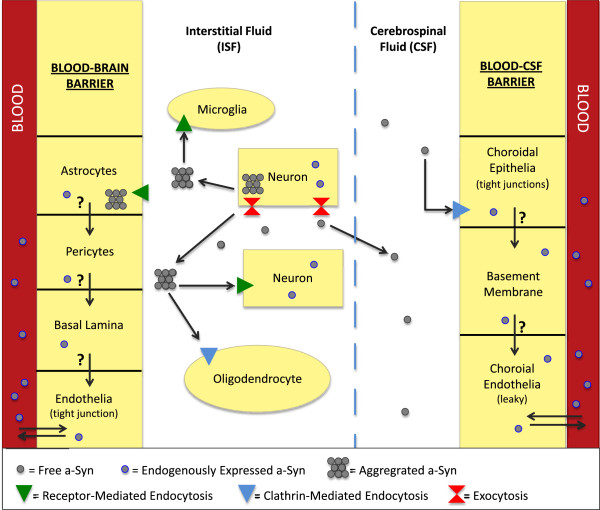
**Proposed disposition of α-Synuclein in neurons, glia, and brain barrier systems.** Both free and aggregated species of a-Syn are transported between neurons or from neurons to various glial cell types. Uptakes of a-Syn species by astrocytes at the BBB and choroidal epithelia at the BCSFB are known to be mediated by the receptor-mediated endocytosis and clathrin-mediated endocytosis, respectively. The influx transport of a-Syn from the blood to the ISF via the BBB or from the blood to the CSF via the BCSFB is currently unknown, neither is the clearance of a-Syn to the blood by either barrier system.

Brain extracellular fluids consist of the CSF in the brain ventricles and interstitial fluids (ISF) between the neurons and glia (Figure 1). The blood–brain barrier (BBB) separates the blood from the ISF and regulates the chemical stability of microenvironment of the brain. The BBB operates as a dynamic boundary between the cerebral vasculature and brain parenchyma, which is highly susceptible to hypoxia, inflammation, endogenous and exogenous insults and other stressors [20]. The BBB consists of (1) endothelial cells comprising the brain capillaries, (2) the basement membrane, (3) the pericytes that surround the endothelia and regulate endothelium inflammation and homeostasis, and (4) the astrocytic glial cells that support the proper functioning of endothelia and provide a direct link between cerebral vasculature and neurons via their unique cap-like structures called end-feet [20-23]. The individual endothelial cells are linked by tight junctions, which are more complex than those of most other vascular endothelia [22,24]. As a result, the paravascular diffusion of hydrophilic molecules that occurs in other endothelia does not occur at the BBB [22,24,25]. The most important role of the BBB is protecting the brain against abrupt fluctuations in blood chemistry (e.g. nutrients, hormones, toxicants, etc.) [24]. Also, the BBB is responsible for the distribution and regulation of the various neurotransmitters used by both the central and peripheral nervous systems [24]. While ionic and hydrophilic molecules are primarily admitted by the BBB via specialized transporters, the permeability of macromolecules and other solutes depends on their size and lipophilicity [22].

The blood-CSF barrier (BCSFB) separates the blood circulation from the CSF (Figure 1) and functions within the highly vascularized choroid plexus located in each of the four brain ventricles [18,19]. The BCSFB is comprised of (1) an apical layer of polarized epithelial cells that face the CSF, (2) supportive connective tissue underlying the epithelial cells, and (3) a basal layer of endothelial cells that faces the blood [18]. The microvilli present on the apical surface of choroidal epithelia increase total surface area of the BCSFB, providing an enhanced capability of uptake by the BCSFB [26]. Furthermore, the relatively large volume of CSF is constantly filtered and secreted by the choroid plexus cells located strategically in the aforementioned ventricles within the brain [19]. The blood flow to the choroid plexus is approximately 5–10 times greater than its flow to other brain regions, which further fortifies the hypothesis of a general cleansing role of the BCSFB [18,26]. Substantial evidence has accrued showing the BCSFB’s ability to clear undesirable contents from the CSF [24-27].

Like the BBB, the tight junctions at the apical layer of the epithelial cells are the structural basis of the BCSFB, which are essential to maintaining barrier function of the BCSFB, due to the naturally leaky and fenestrated endothelium [26]. The CSF is produced by the plexus epithelial cells from water and solutes filtered from the blood [19]. Additionally, the CSF acts as a “sink” for a variety of molecules that can potentially accumulate from the ISF [19]. The contents of the CSF are regulated by the BCSFB as the CSF cycles from the brain, through the BCSFB in the ventricles, throughout the spinal cord, and finally through the subarachnoid space to the blood [19,26]. The CSF, in concert with the BCSFB in the choroid plexus, could potentially carry a-Syn species that are released by neurons to the choroid plexus for clearance.

### α -Synuclein transport by neurons

The increased aggregation of a-Syn found in PD could be the result of 1) the increased production of a-Syn by neural processes, (2) the inter-neuronal transmission of toxic a-Syn aggregated species, 3) the decreased ability for brain to prevent a-Syn from aggregation and against its toxicity, or 4) a combination of the these concepts. a-Syn was originally viewed as an intracellular protein and not capable of being transported out of the cell. Recent studies have provided evidence that contradict this theory. Studies have shown that a-Syn molecules are packaged into vesicles, suggesting that a-Syn can be exported from the cell via exocytosis [15]. Indeed, both monomer and aggregated species of a-Syn are released via exocytosis following intravesicular localization [15]. Furthermore, this process is accelerated in conditions of mitochondrial dysfunction and reduced proteosomal degradation [15], which are both symptoms of PD pathology at the cellular level. Interestingly, the evidence more recently acquired shows that reduced autophagy in neurons also induces the release of a-Syn aggregates via exocytosis; the increased a-Syn exocytosis has been correlated with the increased apoptotic activity in nearby neurons [16]. These findings clearly illustrate the neurons’ capability to eject toxic a-Syn aggregates into the ISF. However, the release of a-Syn aggregates is merely the one half of the cell-to-cell transmission mechanism, which invites the question: what mechanism(s) is/are responsible for the uptake of these extracellular a-Syn aggregates in healthy, neighboring neurons?

The experiment of grafting of healthy neurons with unhealthy neurons possessing a-Syn aggregates can result in a-Syn aggregation in previously healthy neurons [28,29]. A more in-depth study shows that the mechanism underlying the uptake of these aggregates is endocytosis [28]. This finding confirms that a-Syn aggregates can be transported inter-cellularly via exocytosis and endocytosis. Other researchers have provided additional evidence that supports endocytosis as a mechanism for a-Syn aggregate uptake [30]. It is also believed that a-Syn is taken up by neurons via different mechanisms depending on the species of a-Syn. Monomer a-Syn can diffuse across the membrane in a passive manner, while the aggregated species are taken up by endocytosis, which is thought to be receptor-mediated [30]. Interestingly, the findings from this group have also suggested that neurons take up a-Syn aggregates in an attempt to degrade the inclusions while sparing a-Syn monomers from degradation [30]. Due to the aforementioned compromised autophagy in neurons afflicted with a-Syn aggregates, these inclusions are free to induce further intracellular damage including the extension of a-Syn polymerization. Additionally, a more recent study indicates that extracellular a-Syn aggregates can induce lysosomal rupture following uptake via cell-to-cell transmission [31]. Therefore, the aggregates may attack the mechanisms required for their clearance in neurons while simultaneously exacerbating intracellular toxicity.

At the cellular level, PD pathology is initiated when a-Syn aggregates are unable to be degraded by the afflicted neurons, which facilitates their export from the cell via exocytosis. These extracellular polymers then promote this vicious cycle in neighboring cells after being taken up from the ISF via endocytosis. These inclusions wreak additional havoc within the cell by inducing lysosomal rupture, mitochondrial dysfunction, continued a-Syn aggregation, and eventual apoptosis while simultaneously extending this cycle of pathology [9,17,31,32]. This pathology can be combated with therapeutic strategies including reversing the aggregation of a-Syn and enhancing the cells’ ability to degrade these polymers. One potential therapeutic strategy would be to strengthen the ability of the barriers to clear these aggregates from the brain, so that they can be degraded elsewhere in the body. This strategy cannot be employed without a fundamental understanding of a-Syn transport and regulation by these barriers.

### α-Synuclein transport by astrocytes: implications in BBB transport

Astrocytes are an integral part of the BBB (Figure 1). The nature of the BBB in regulating which molecules are accessible to the brain, places the system in an ideal position to assist in the uptake and clearance of a-Syn between the ISF and blood. The BBB may possess a therapeutic advantage over the BCSFB because of its immediate contact with neurons that border cerebral vasculature via the astrocytes [33]. Consequently, any clearance of neuronal-derived a-Syn by the BBB would likely begin with the astrocytes, and thus the ability of astrocytes to transport a-Syn aggregates to the endothelial cells is critical to the release of a-Syn to the blood (Figure 1). Previous research, however, shows that the BBB potentially does more harm than good in attempts to clear toxic a-Syn species from neurons and the ISF. Initial evidence has shown the presence of aggregated a-Syn species in astrocytes of post-mortem PD patients [34,35]. However, the research does not provide any evidence pertaining to the mechanisms behind the appearance of these accumulations in astrocytes. Interestingly, a hypothesis has since been developed that these a-Syn inclusions originate from outside the cell and are being absorbed by the astrocytes [35]. Another research group later confirmed this hypothesis; their research shows that astrocytes acquire extracellular a-Syn aggregates via endocytosis [36,37]. In a set of experiments, Lee and colleagues have shown that rat primary astrocytes take up a-Syn aggregates (1) from conditioned medium containing a-Syn aggregates collected from differentiated SH-SY5Y cells and (2) when co-cultured with SH-SY5Y cells that overexpress a-Syn. Astrocytes transfected with a dynamin-1 K44A mutation, which prevents endocytosis via the inhibition of endocytic vesicle formation, show a significant decrease in a-Syn uptake [37]. These results confirm the primary mechanism of a-Syn uptake is by endocytosis. In addition, this group of investigators has also shown that glial a-Syn accumulation occurs in a-Syn overexpressing transgenic (tg) mice compared to APP tg and non-tg control mice, where a-Syn is present only in neurons [37]. Finally, this group has shown that a-Syn glial accumulation induces pro-inflammatory gene expression and cytokine production, suggesting that the uptake of extracellular a-Syn can lead to cellular toxicity in astrocytes [37].

It must be emphasized that the astroglial a-Syn toxicity appears to be very similar to the a-Syn pathology pathway seen in neurons including the death of afflicted astrocytes. Another factor to consider with respect to a-Syn clearance by the BBB is the endogenous expression of a-Syn by this barrier. The endogenous expression of a-Syn in astrocytes has recently been illustrated in primary cultures of rat astrocytes [37]. Therefore, we can postulate that extracellular toxic a-Syn species that enter astrocytes could generate new a-Syn oligomers and fibrils in astrocytes, which would also contribute to cell stress and death. The similarity between a-Syn dysfunction in neurons and astrocytes alludes to the inability of the BBB to degrade or clear toxic a-Syn species from brain. Interestingly, the similarities between the a-Syn pathologies of neurons and astrocytes also suggest, albeit remotely, that reversing a-Syn aggregation may be an effective therapeutic strategy in neurons, astrocytes, and other glial cell types. If the BBB is to be utilized therapeutically to clear toxic a-Syn species from the brain, astrocytes should become a prime therapeutic target for their role of the first contact with a-Syn in brain parenchyma. Furthermore, the expression and behavior of a-Syn in other cell types of the BBB must be investigated. For example, endothelial cells from human cerebral blood vessels have been shown to express a-Syn mRNA in previously-published literature [38]. More investigation in the expression and transport of a-Syn species by all cell types of the BBB is needed in order to fully understand the potential for a-Syn pathology throughout the BBB and establish any potential therapeutic strategies involving this barrier.

### α-Synuclein transport by choroidal epithelia: implications in BCSFB clearance

While there is still much unknown about a-Syn transport by the BBB, even less is known about the role of the BCSFB in regulating a-Syn in the CSF. The BCSFB is capable of clearing materials from the brain; but little information in the current literature suggest a potential pathway for the BCSFB to clear toxic a-Syn species present in the CSF. Additionally, the extent to which the BCSFB produces endogenous a-Syn was unknown. A clear difference between the BBB and BCSFB is that the BCSFB does not have a direct cellular link to neurons. Rather, the BCSFB plays a role in brain homeostasis via the regulation of the CSF that surrounds and immerses brain parenchyma. This raises the question of whether this position of the BCSFB provides an advantage in the clearance of toxic a-Syn species or whether it places the BCSFB at an even greater risk of a-Syn-induced damage compared to the BBB.

It has been suggested that most of the a-Syn found in the brain and CSF is produced by neurons and/or neuroglia [39]. Yet, a smaller, secondary source of a-Syn may originate from transport from the blood to CSF (Figure 1). Naturally, this raises the question as to how the BCSFB in the choroid plexus regulates a-Syn in the CSF, if at all. Recent longitudinal studies have shown the blood concentration of a-Syn can be as high as about 3.5 μg/mL [40]. This is over 1000-fold higher in comparison to the CSF [40-42]. There is also evidence showing that a-Syn mRNA is expressed by erythrocytes [43,44]. However, it remains unknown whether the fraction of a-Syn content in blood originates from organs other than the brain or is derived from the CNS. The exponentially higher a-Syn level found in the blood compared to CSF suggests that the BCSFB must actively maintain a-Syn levels in the CSF against a sharp concentration gradient between the blood and the CSF. Therefore, if the BCSFB could clear a-Syn from the CSF, it must perform this function via some type of energy-dependent active transport in order to transport a-Syn against this concentration gradient. While the presence of a-Syn in the CSF has been confirmed by previous research [39,41,42], the actual transport of a-Syn across the BCSFB and the mechanism(s) involved are currently unknown. The evidence of intracellular and cell-to-cell a-Syn transport via endocytosis in neurons and astrocytes [28,30,45] suggests that a-Syn could be transported by endocytosis at the BCSFB.

**Figure 2:**
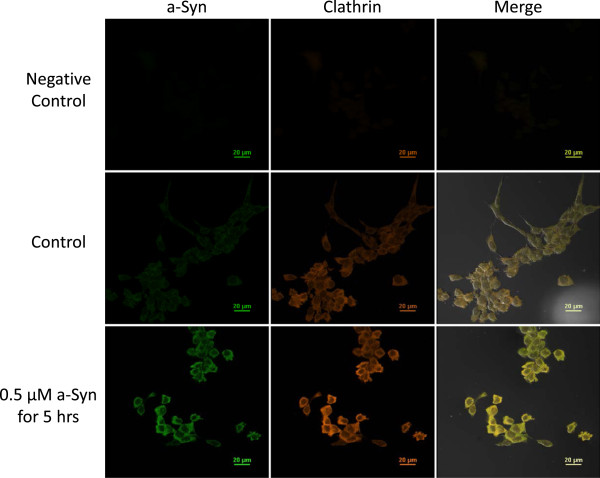
**Expression of α-Synuclein and clathrin in Z310 cells with or without a-Syn treatment in a typical experiment (n = 5).** Z310 cells are immortalized rat choroidal epithelial cells established in this laboratory [47]. The cells were treated with or without 0.5 μM recombinant human a-Syn for 5 hours prior to immunostaining with the primary antibodies, followed by fluorescent secondary antibodies. Fluorescent signals for a-Syn and clathrin are shown in green and red, respectively. Merged images show a co-localization of a-Syn signals with those of clathrin.

Recent data from this laboratory have identified the presence of the mRNA encoding a-Syn as well as a-Syn proteins in choroid plexus tissue collected from rats; similar expression of a-Syn was also found in rat choroidal primary cells as well as in an immortalized rat choroid epithelial Z310 cell line (Figure 2) [45,46]. We suspect that this expression of a-Syn may play a role in a-Syn pathology and contribute to the development of a-Syn aggregates. Similar to the pathological pathway in astrocytes, the BCSFB may attempt to clear a-Syn aggregates from the CSF via the uptake of these polymers, which provides an opportunity for these polymers to interact with endogenous a-Syn in cells of the BCSFB and promote cell stress and accelerated a-Syn pathology.

Recent studies from this laboratory also show that the BCSFB is capable of taking up a-Syn molecules from the CSF. When immortalized Z310 rat choroidal epithelial cells were stained for a-Syn, the expression of naturally existing a-Syn in control Z310 cells was evident (Figure 2) [46]. Furthermore, by incubating the cells with 0.5 μM a-Syn for 5 hours, an increased fluorescent signal in the cells became evident; this suggests that the BCSFB cells have the ability to take up a-Syn from the extracellular medium [46]. The cells were also simultaneously stained for clathrin, a protein that forms the coated vesicles and nonspecifically transports large molecules into the cells by endocytosis [48]. The immunocytochemical data clearly showed that the fluorescent signals of a-Syn were co-localized with signals from clathrin (Figure 2). More interestingly, the clathrin-fluorescent signal increased following a-Syn treatment with respect to controls, indicating an up-regulated expression of clathrin in response to the presence of external a-Syn. These data suggest that clathrin-mediated endocytosis appears to be, at least partially, responsible for a-Syn uptake in choroidal Z310 cells [46].

More recently, we investigated the transport of monomer a-Syn across the BCSFB via an *in vitro* model that utilizes rat primary choroid plexus cells cultured on a permeable membrane of a Transwell chamber [49]. Our results showed that after 24 and 48 hours of incubation with a-Syn from the apical side of the barrier, the BCSFB monolayer was capable of establishing and maintaining an a-Syn gradient, with a significant majority of the a-Syn on the basal side of the barrier, i.e. the blood side [49]. These data clearly show that the BCSFB is capable of transporting a-Syn between the blood and the CSF. Our observations may provide a foundation for understanding the role of the BCSFB in a-Syn transport between the CSF and the blood. This enables future avenues of research to be explored including (1) the BCSFB’s ability to clear toxic a-Syn species from the CSF, (2) the mechanism(s) behind the uptake of a-Syn by the BCSFB, and (3) the role, if any, endogenous expression of a-Syn might play in potential a-Syn-related pathological pathways at the BCSFB. Understanding the relationship between a-Syn and the BCSFB is essential in order to ascertain the BCSFB’s potential role in PD pathology. Also, understanding this phenomenon could assist with understanding other neurodegenerative disorders, clarify the potential of a-Syn in the CSF as a reliable biomarker for PD, and assess the BCSFB’s potential as a therapeutic target for PD.

### Toxicological implication of a-Syn transport at brain barriers

There is an abundance of factors involving the progression of neurodegeneration that have yet to be reported in relation to a-Syn transport by the blood–brain barriers. Most cases of sporadic PD are considered to be idiopathic, but it is generally believed that the disease is a result of unknown environmental factors [49]. Recent evidence has suggested that some of these cases may be related to the exposure to environmental factors including heavy metals and pesticides [50,51]. Exposure to such factors could have significant effects on a-Syn regulation by both barriers and possibly contribute to PD pathology. For example, our lab has shown that toxic manganese exposure can induce aggregation and altered uptake of a-Syn in rat primary choroid plexus cells within 2 hours [49]. In addition, there are many genetic mutations in proteins other than a-Syn that contribute to a-Syn toxicity and PD development [52,53]. The G2019S LRRK2 mutation, the most common mutation found in familial PD phenotypes, has been showed to interact with a-Syn during chaperone-mediated autophagy and consequently, promote a-Syn dysfunction [54]. These and various other factors could likely play critical roles in a-Syn regulation by the BBB and BCSFB from the standpoint that these factors are detrimental to brain health and must be countered effectively. As research in these areas progresses, a new answer may present itself that will allow us to use the relationship between the blood–brain barriers and a-Syn therapeutically.

## Conclusions

The current understanding of a-Syn transport by various cell types in the BBB and BCSFB as well as in neurons is summarized in Table 1. In general, the amount of investigation into the relationship between the blood–brain barriers and a-Syn pathology in PD remains miniscule. Consequently, very little is known about these relationships. With the limited information, we propose a tentative scheme of a-Syn disposition in brain and further suggest that pertinent research is needed to advance the field (Figure 1). For a-Syn transport from blood to ISF via the BBB, it is currently unknown whether brain endothelial cells possess the receptor-mediated or clathrin-mediated endocytosis, which could take up a-Syn molecules from the systemic circulation and serve as the source of a-Syn in brain parenchyma. We also do not know how a-Syn is transferred from cerebral endothelia to astrocytes prior to reaching neurons, or *vice versa*, from astrocytes to endothelia. These interesting questions could serve well as the research objectives for understanding a-Syn transport by the BBB.

**Table 1:** Studies and findings on the uptake and release of a-Syn by neurons, glia, and brain barrier cell types

**Cell type**	**a-Syn transport**	**References**
**Blood–brain barrier (BBB)**	1) Astrocytes: Endogenously expresses a-Syn. Astrocytes take up a-Syn by endocytosis; inflammatory activation occurs upon uptake of a-Syn aggregates. Astrocytes also secrete free a-Syn.	Braak *et al*., 2007 [3]
	2) Endothelia: endothelia of cerebral blood vessels express a-Syn endogenously. No detectable expression of a-Syn was found in BBB endothelia.	Kim *et al*., 2008 [23]
	3) Perictyes: Unknown	Lee *et al*., 2010a [35]
	4) Basal Lamina: Unknown	Lee *et al*., 2010b [36]
		Kim *et al*., 2013 [37]
		Tamo *et al*., 2002 [38]
**Blood-CSF barrier (BCSFB)**	Choroid Epithelia: Immortalized Z310 cells express a-Syn endogenously. Z310 cells uptake free a-Syn; clathrin is upregulated during a-Syn exposure. Primary CP epithelia from rat express a-Syn endogenously and take up free a-Syn.	Bates *et al*., 2012 [46]
		Bates *et al*., 2013 [49]
**Neurons**	Neurons are capable of both the uptake and secretion of a-Syn. Free and aggregated a-Syn can be secreted and taken up by neurons. Cell-to-cell transmission can occur between neurons or with multiple glial types (e.g. astrocytes, microglia, etc.)	Lee *et al*., 2005 [15]
		Lee *et al*., 2013 [17
		Desplats *et al*., 2009 [28]
		Lee *et al*., 2008a [30]
		Freeman *et al*., 2013 [31
		Büchel *et al*., 2013 [32]
**Glia (excl. astrocytes)**	1) Microglia: Microglia take up free and toxic a-Syn aggregates from interstitial fluid. Inflammatory activation upon uptake of a-Syn.	Wakabayashi *et al.,* 2000 [33]
	2) Oligodendrocytes: Uptake of aggregated a-Syn was shown to be clathrin-dependent. Consequently, intracellular inclusions containing a-Syn can occur	Kisos *et al.,* 2012 [55]
		Lee *et al*., 2008b [56]

Within brain parenchyma, the ultimate fate of a-Syn, specifically toxic, aggregated species, following the release from neurons (and other cell types), remains elusive. Neurons are known to produce a-Syn, and by exocytosis release free and/or aggregated a-Syn species to the ISF, which results in the exposure of toxic a-Syn species to neighboring neurons and glia. Most of the neural cell types are capable of degrading free or small, oligomeric a-Syn species; thus, the influx of these a-Syn aggregate species could potentially start the intracellular, pathological cycle of a-Syn aggregation, cell damage and death, and the eventual release of additional toxic forms of a-Syn to nearby cells [36,37,55,56]. Astrocytes, for example, can absorb these polymers via receptor-mediated endocytosis. This could lead to cellular injury and apoptosis, which may also compromise the BBB to some degree. Accumulation of a-Syn in astrocytes would logically lead to the assumption that a-Syn may be transported out of brain by the BBB. However, there is no report in literature showing evidence to substantiate this hypothesis.

Recent data have provided the evidence of endogenous a-Syn expression in the choroidal epithelial cells in the BCSFB and the ability of the BCSFB to transport a-Syn across a primary BCSFB monolayer *in vitro*. This finding can be used as a launching point for future investigations to further explore the relationship between a-Syn, the BCSFB, the external factors that can induce a-Syn dysfunction at the BCSFB, and how that dysfunction can alter the physiology and function of the BCSFB. We propose that under normal conditions, the BCSFB can clear a-Syn from the CSF by degrading the protein within the barrier cells and/or transporting the protein to the blood. Should the BCSFB be unable to clear a-Syn from the CSF, the implication of BCSFB as a contributing factor to a-Syn-associated injury cannot be ignored.

Overall, findings discussed in this article have described the uptake of a-Syn by astrocytes and by choroidal epithelial cells, but these findings are merely the first step to understand the potential clearance or degradation of a-Syn by the BBB and BCSFB. Therefore, substantial research remains to be done in order to ascertain the ability of the brain barrier systems to regulate a-Syn in the brain. This line of research will contribute significantly to our understanding of synucleinopathies, particularly in PD, and identification of new therapeutic targets.

## Abbreviations

a-Syn: α-Synuclein; BBB: Blood–brain barrier; BCSFB: Blood-cerebrospinal fluid barrier; CSF: Cerebrospinal fluid.

## Competing interests

The authors declare that there are no financial or non-financial competing interests.

## Authors’ contributions

CB searched literature, conducted experiments and wrote the manuscript. WZ outlined, commented and revised the manuscript. Both authors have read and approved the final version of the manuscript.
